# Regulation of metabolism by a neuroendocrine FGF signaling pathway

**DOI:** 10.1186/2049-3002-2-S1-O17

**Published:** 2014-05-28

**Authors:** David J Mangelsdorf, Steven A Kliewer

**Affiliations:** 1 Department of Pharmacology, University of Texas Southwestern Medical Center, Dallas, TX 75390, USA; 2 Howard Hughes Medical Institute, USA; 3 Department of Molecular Biology, University of Texas Southwestern Medical Center, Dallas, TX 75390, USA

## 

Fibroblast growth factor 21 (FGF21) is a hepatokine that during fasting (in the absence of insulin and dietary nutrients) plays an important role in the adaptive response to starvation. However, in the setting of obesity and nutrient excess, FGF21 has potent pharmacologic effects that include weight loss and improved insulin sensitivity due in large part to FGF21’s ability to mount a robust thermogenic response. FGF21 is also a target gene of PPARα and PPARγ agonists, and mediates many of the effects of these agonists in liver and adipose tissue. Although these effects make FGF21 an attractive therapeutic target for obesity, FGF21 also inhibits growth, causes bone loss, and suppresses female reproduction. Interestingly, many of the pharmacologic effects seen in obese mice are also part of the adaptive physiologic response seen in starvation. To understand the anatomical sites of FGF21 action, we have generated whole-body and tissue-selective knockouts of β-Klotho, the co-receptor believed to be required for FGF21 signaling. All of the known effects of FGF21 are lost in β-Klotho knockout mice. Selective elimination of β-Klotho in a number of different tissues, including adipose, liver, and particularly in the CNS has revealed the existence of a complex peripheral and neural endocrine circuit, which coordinates the diverse physiologic and pharmacologic actions of FGF21.

